# Patient decision aids for antidepressant use in pregnancy: a pilot randomised controlled trial in the UK

**DOI:** 10.3399/bjgpopen19X101666

**Published:** 2019-12-11

**Authors:** Hind Khalifeh, Emma Molyneaux, Ruth Brauer, Simone Vigod, Louise M Howard

**Affiliations:** 1 Senior Clinical Researcher and Consultant Perinatal Psychiatrist, Section of Women’s Mental Health, Institute of Psychiatry, Psychology and Neuroscience (IOPPN), King’s College London, London, UK; 2 Research Associate and Honorary Lecturer, Section of Women’s Mental Health, Institute of Psychiatry, Psychology and Neuroscience (IOPPN), King’s College London, London, UK; 3 Lecturer Pharmacoepidemiology, School of Pharmacy, University College London, London, UK; 4 Associate Professor and Psychiatrist-in-Chief, Women’s College Hospital and Women’s College Research Institute, and Department of Psychiatry, University of Toronto, Toronto, Canada; 5 Professor of Women's Mental Health, Section of Women’s Mental Health, Institute of Psychiatry, Psychology and Neuroscience (IOPPN), King’s College London, London, UK

**Keywords:** pregnancy, depression, antidepressants, patient decision aid, pilot, randomised controlled trial

## Abstract

**Background:**

Decision-making regarding antidepressant use in pregnancy is challenging, given the uncertain evidence base on the benefits and risks for women and their children. Patient decision aids (PDAs) can improve shared decision-making for complex health decisions but no evidence-based PDAs exist for antidepressant use in pregnancy.

**Aim:**

To assess the feasibility of a full-scale randomised controlled trial (RCT) to evaluate the efficacy of an electronic PDA on antidepressant use in pregnancy.

**Design & setting:**

A UK-based pilot parallel-group RCT.

**Method:**

The study recruited women whose clinicians recommended an antidepressant for depression in a current or planned pregnancy, and who were uncertain about antidepressant use while pregnant. Women were recruited via clinician or self-referral, and randomised to online access to the PDA or online access to standard resource list, with primary follow-up at 4 weeks and longer-term follow-up. The primary outcome was protocol feasibility (recruitment target of 50 women and follow-up rate of 80%). Outcome measures for a future full-scale RCT included the decisional conflict scale (DCS).

**Results:**

Fifty-one women were recruited with a follow-up rate of 90.2% at 4 weeks. The PDA received good overall satisfaction ratings (mean 4.2/5). Analysis of covariance (ANCOVA) indicated a small improvement in decisional conflict at 4 weeks, accounting for baseline scores (DCS regression coefficient = -3.5, 95% confidence intervals [CI = -12.6 to 5.6]).

**Conclusion:**

This pilot RCT for an electronic PDA on antidepressant use in pregnancy showed that the study protocol was feasible, with high rates of participant satisfaction among those randomised to the PDA.

## How this fits in

The decision regarding whether or not to use antidepressants in pregnancy is clinically challenging. Patients and clinicians making this decision need to weigh up relative benefits and risks for mother and baby using an uncertain evidence base. PDAs improve shared decision-making for such complex clinical decisions, but no relevant evaluated PDAs exist. This pilot RCT of an electronic PDA for antidepressant use in pregnancy showed that the trial protocol was feasible, with high patient satisfaction, supporting a future full-scale clinical trial.

## Introduction

Depression is one of the commonest morbidities of pregnancy, affecting around 10–15% of pregnant women.^1^ Untreated or incompletely treated depression is associated with potential adverse effects for mother and unborn child, including prematurity, childhood emotional and behavioural problems, and, rarely, maternal suicide.^1,2^ Antidepressants are indicated for the treatment of moderate-to-severe depression in pregnancy when psychotherapy alone is unlikely to result in substantive improvements.^3^ There is evidence that the proportion of women taking antidepressants during pregnancy has increased substantially in recent years;^4^ for example from 2% in 1991–1992 to 13% in 2012–2016 in a two-generational study of young mothers in southwest England.^5^

National Institute for Health and Care Excellence (NICE) guidelines recommend a stepped-care approach to the assessment and management of perinatal depression, with first-line management in primary care.^6^ This includes first-line management of antidepressant use in pregnancy, in consultation with experts where necessary.^6^ When considering antidepressant use in pregnancy, women and their clinician, usually their GP, have to weigh the risks of ongoing or relapsing depression versus the potential risks of antidepressant use, in the face of scientific uncertainty.^7^ Recent studies have identified key barriers that women face to decision-making in this context, which include lack of information, difficulty in weighing up risks and benefits, unhelpful external influences, and a desire for greater involvement in the decision-making process.^8,9^ The difficulties that GPs face around antidepressant prescribing in pregnancy have also been highlighted, including conflicting sources of information, some women’s reluctance to take medication, and the importance of empowering women to be involved in decisions about their care.^10^

PDAs aim to improve shared decision-making for complex health decisions which involve weighing uncertain benefits versus uncertain harms.^11^ Therefore, they may be a valuable tool for improving decision-making about antidepressant use in pregnancy. NICE guidelines on good practice in shared decision-making endorse the use of PDAs where suitable, high-quality aids are available; however there is no previously evaluated PDA for antidepressant use in pregnancy. Recently, an electronic PDA for antidepressant use in pregnancy was developed in Canada, by experts in decision aids, perinatal psychiatry, and psychopharmacology.^12^ The study reported here was a pilot RCT of this PDA, adapted for the UK setting, to assess the feasibility of conducting a full-scale RCT to evaluate its efficacy in the UK. The objectives of this pilot RCT were:

1. to assess feasibility in terms of recruitment, retention, and adherence to trial protocol;
2. to assess the acceptability of the intervention; and
3. to estimate preliminary effect sizes of important clinical outcomes, such as decisional conflict.

## Method

The PDA was developed in Canada, guided by the International Patient Decision Aids Standard collaboration guidelines;^13^ relevant published systematic reviews on the benefits and harms of antidepressant treatment in pregnancy;^14–17^ and evidence related to the decisional needs of women considering this complex decision (including qualitative and quantitative data from interviews with women facing this decision).^8,9^ The PDA was adapted for the UK setting by making minor changes to its content (for example, removing references to costs of treatment and to medications that are not licensed for use in the UK).

### Study design, setting, and participants

A pilot parallel-group, investigator-blind randomised controlled trial was conducted with a 1:1 allocation ratio to active intervention arm (access to electronic patient decision aid) or control arm (access to electronic information sheet). Recruitment through clinical services was initially restricted to services in south east London (via GP practices, mental health services, and antenatal maternity and obstetric services) and later expanded to include three maternity services in northern and south east England. Recruitment was supplemented by national advertisements on relevant websites and social media (for example the National Childbirth Trust, Tommy’s Charity, and PANDAS Foundation), and direct advertising on Facebook. Women could self-refer to the study via phone, email, or online form. Researchers carried out initial eligibility checks, and then with the woman’s consent contacted the clinician advising her on antidepressant use to establish eligibility and inform them of the woman’s planned participation in the trial. Women gave written consent before participation. Inclusion and exclusion criteria are summarised in Box 1.

Box 1 **Participant inclusion and exclusion criteria**

Women were eligible to participate if they met all of the following inclusion criteria:

- Aged 18 or over
- Planning a pregnancy or ≤30 weeks pregnant at enrolment
- Had been offered to start or continue an SSRI or SNRI antidepressant as treatment for depression by their clinician
- Had moderate-to-high decisional conflict (score of >25 on the Decisional Conflict Scale)
- Gave written informed consent to participate

Women were excluded from the study if they met any of the following exclusion criteria:

- Had had alcohol or drug abuse or dependence in the previous 12 months
- Had active suicidal ideation or psychosis
- Were incapable of consenting to participation
- Had major obstetric complications (that could influence guidance about antidepressant use) or foetal cardiac anomaly in the current or in a past pregnancy
- Were visually impaired or did not have sufficient English language proficiency to use the PDA

### Interventions

Experimental and control group interventions were delivered online. Women randomised to the control arm had access to a printable resource sheet containing references to standard published information on depression and antidepressant use in pregnancy. Women randomised to the intervention arm had access to the electronic PDA, as well as the printable resource sheet. All participants received clinical monitoring as part of their standard care. The interactive, electronic PDA had three sections. Section 1 provided evidence-based information about depression during pregnancy, and about treatment options as recommended in national guidance. This included information on the epidemiology and risk factors for antenatal depression, as well as information on the range of recommended treatments (including psychological therapy and antidepressants), their indications, and how they are implemented in clinical practice. Section 2 provided evidence-based information on the risks and benefits of non-medicated depression and of antidepressant use. The information about antidepressant use was focused on selective serotonin reuptake inhibitors (SSRIs). This section included information on the risk of relapse with and without medication use, side effects of antidepressants for the mother, and the effects of prenatal depression and antidepressant use on neonatal and child outcomes (for example prematurity, growth abnormalities, and neurodevelopmental problems). The PDA was designed to provide women with brief information about risks or benefits, with each statement followed with a link that women could click for more information about the evidence behind this statement. Women could choose whether they wanted to access this additional information. If the literature on specific benefits and risks was particularly unclear, this was stated. This section also included questions to help women clarify the relative importance of different benefits and risks to them (using a sliding scale visual aid), as well as the influence of others (for example, family, friends, or professionals) on their decision-making process (using a traffic light visual aid). Section 3 provided a summary of the information reviewed on risks and benefits, the participant’s rating of their relative importance and the participant’s perception of external influences on their decision-making process. Participants could print this summary and use it in clinical follow-up (with any clinician who is advising them on antidepressant use in pregnancy). Women could access the study website as many times as they wanted, using laptops, desktops, smartphones, or tablets; and they had access to the website up to the end of the study period.

### Study procedures

Interviews were conducted in person or over the phone. After completion of baseline measures (t0), participants were emailed their login details and asked to access the study website before their next appointment with their clinical provider (recommended within 4 weeks of randomisation). Participants’ login details gave them access to the decision aid or the online resource sheet, dependent on which group they had been randomly allocated to. Follow-up outcome measures were collected at 4 weeks post-randomisation (t1) and either at 12 weeks postpartum (if the participant enrolled while pregnant) or 6 months post-randomisation (if the participant enrolled while planning a pregnancy) (t2). Clinicians whose patients were randomised to the electronic PDA were asked to complete a provider perspective survey at the end of the study period.

### Study measures

Feasibility was assessed by measuring eligibility rate, recruitment rate, non-participation reasons, and follow-up rates. Acceptability was measured using study-specific questionnaires for participants and clinicians (each including Likert scale items with a range of 1–5 and open-ended questions). Clinical effectiveness was assessed by estimating preliminary effect sizes for the following outcomes:

1. decisional conflict, measured at baseline and t1 using the DCS, a 16-item self-report scale (score range 0–100), with scores of ≥25 indicating decisional uncertainty);^18^
2. knowledge of depression treatment options, measured at baseline and t1 using a study-specific questionnaire;^12^
3. depressive symptoms, measured at baseline, t1, and t2 using the Edinburgh postnatal depression scale (EPDS), a 10-item self-report depression screening measure (score range 0–30);^19^ and
4. anxiety symptoms, measured at baseline, t1, and t2 using the Spielberg state-trait anxiety inventory (STAI), a 40-item self-report measure (score range 20–80).^20^
  Baseline measures included sociodemographics, obstetric and psychiatric history, and an interviewer-administered standardised assessment of psychiatric disorders using a shortened version of the mini international neuropsychiatric interview (MINI, version 5.0),^21^ which maps onto DSM-IV & ICD-10 diagnoses (modules on affective, anxiety, psychotic and substance misuse disorders, and suicidality were included).

### Sample size

It was estimated that a minimum sample size of 40 (20 per intervention arm) was required for this pilot RCT, allowing assessment of the acceptability of the intervention.^22^ The study aimed to recruit at least 25 participants to each intervention group, allowing for 20% loss to follow-up. As this is a pilot RCT, it was not expected to be able to detect a statistically significant difference between groups in clinical outcomes with this sample size.

### Randomisation and blinding

Participants were randomised in a 1:1 ratio using a computer-generated random allocation sequence that was activated at first login to the study website, with stratification by whether they were recruited from primary care, maternity care, or psychiatric settings. Researchers were blind to group allocation at all data collection time points. Participants were most likely able to identify whether they had been randomised to the PDA, as this multistage interactive tool was clearly different from the single page resource sheet (control condition). Therefore, they could not be blinded as to which intervention they received, but were asked not to disclose this to the researcher at follow-up. Health providers may or may not have been blinded, depending on whether patients discussed the intervention with them, but they were not involved in data collection.

### Analysis

Descriptive statistics were used for the feasibility and acceptability outcomes. In addition, responses to open-ended questions in the acceptability questionnaires were collated. Clinical effectiveness measures were compared between the control and intervention arms using a one-way ANCOVA model, where the covariate was the baseline score. Effect sizes were estimated using the partial eta squared method (calculated using ANCOVA post-estimation commands in STATA, version 13). The intention-to-treat principle was used and participants with missing follow-up data were excluded from the analysis and treated as lost to follow-up.

## Results

**Figure 1.**
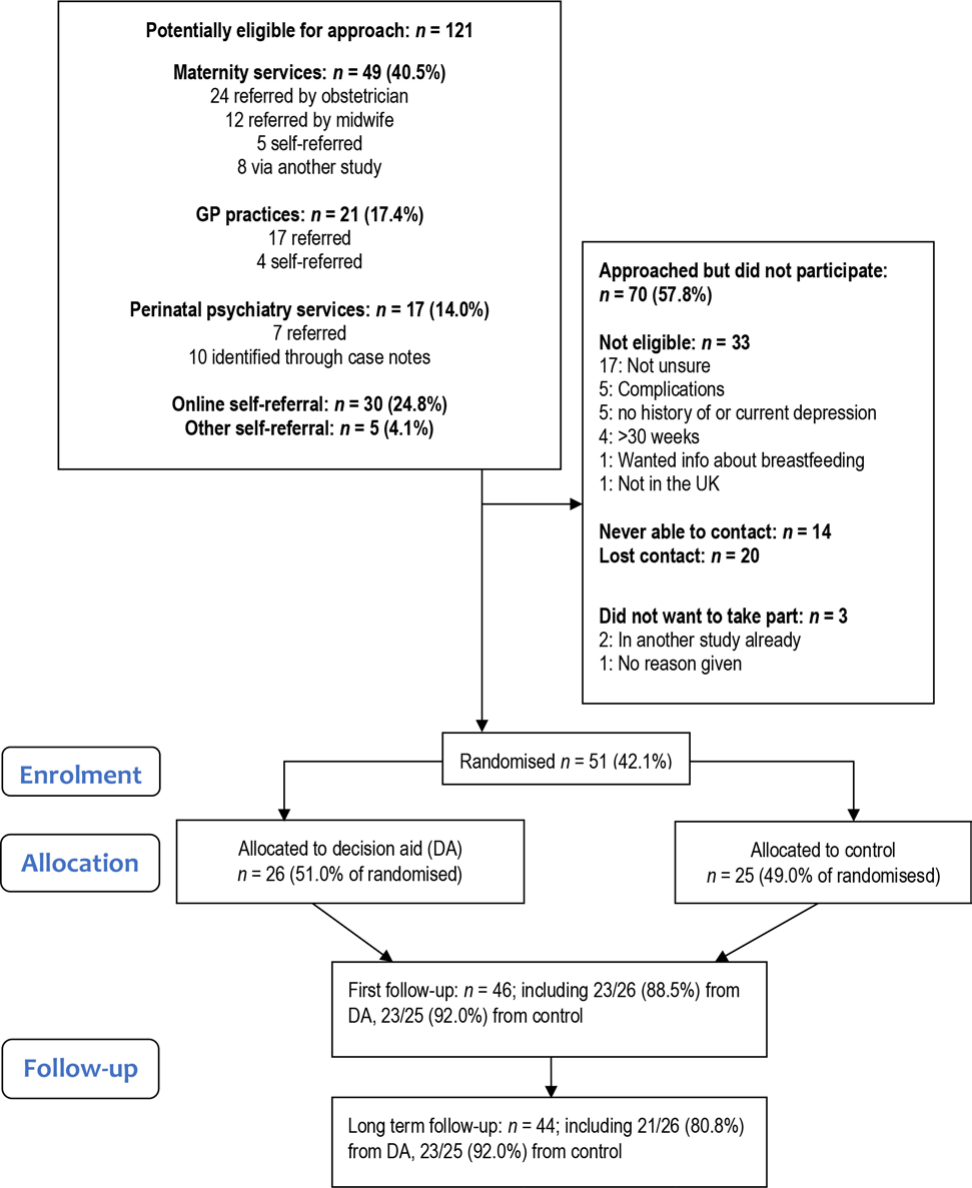
Participant flow diagram

The participant flow diagram is shown in Figure 1 and baseline characteristics are shown in Table 1. In total, 121 potentially eligible women were identified, and 51 women were enrolled in the study (in July 2015–February 2017); 26 in the intervention arm and 25 in the control arm. Recruitment ended when target was reached. At baseline, 47.1% of women met diagnostic criteria for current major depression and 68.6% had a current anxiety disorder. The clinician advising the woman on antidepressant use was most commonly a GP (76.5%), and less commonly an obstetrician (18.6%), or a psychiatrist (5.9%).

**Table 1.** Sample characteristics

Variable	Level	PDA, *n* (%)(*n* = 26)	No PDA, *n* (%) (*n* =25)
**Socio** **demographic** **data**			
Pregnancy status	Planning	9 (34.6)	12 (48.0)
	Pregnant	17 (65.4)	13 (52.0)
Mean age, years (SD)	31.7 (4.5)	34.0 (5.9)
Marital status	Married or cohabiting	23 (92.0)	23 (88.5)
Education	College/university	14 (53.8)	21 (84.0)
Family income (≥£40 000)		14 (53.8)	18 (72.0)
Obstetric history	≥1 prior births	16 (61.5)	10 (40.0)
Antidepressants	Prior but no current use	12 (46.1)	6 (24.0)
	Current use	14 (53.9)	19 (76.0)
**Psychiatric disorders (** **MINI** **)** **^a^**			
Current disorders	Any disorder	21 (80.8)	17 (68.0)
	Major depression	15 (57.7)	9 (36.0)
	Anxiety	20 (76.9)	15 (60.0)

^a^The abbreviated Mini-International Neuropsychiatric Interview, including modules for mood disorders, anxiety disorders, and substance misuse disorders.

PDA = patient decision aid. SD = standard deviation.

### Feasibility, usage, and acceptability

Feasibility in terms of eligibility and retention is summarised in Figure 1, demonstrating feasibility of reaching the recruitment target and high follow-up rates (90.2% at t1 and 86% at t2). The initial recruitment strategy focused on local clinical services was ineffective with a slow recruitment rate (one patient recruited over 6 months), and this was resolved by expanding recruitment sites to three additional maternity services in northern and south east England, and via direct advertising to potential participants nationally via relevant websites (with a subsequent recruitment rate of around one participant per week).

**Figure 2.**
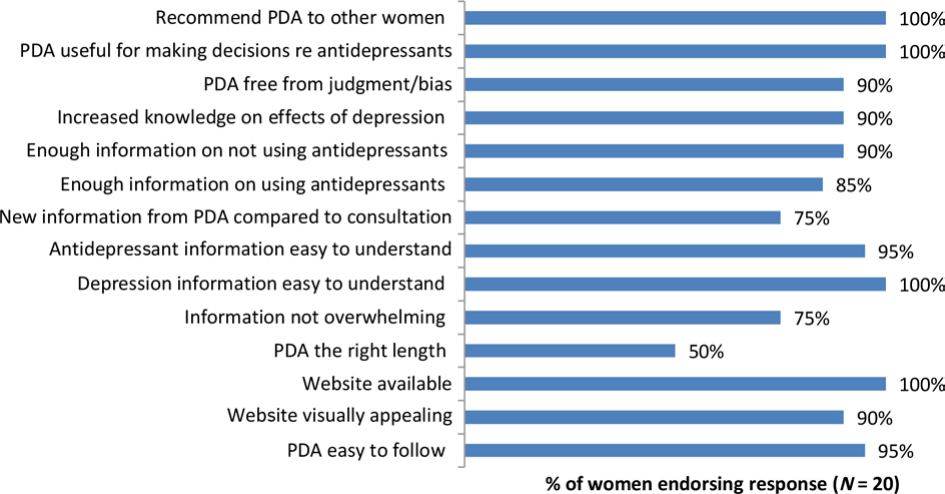
Responses to acceptability questionnaire among women who were randomised to receive the patient decision aid (PDA)

Usage data was collected by the company hosting the online PDA tool. Of the women randomised to the intervention arm, 88.5% (*n* = 23) accessed the PDA tool online within 4 weeks of randomisation. All the women who accessed the tool completed section 1, and more than 80% completed sections 2 and 3. In terms of acceptability, participants allocated to the PDA intervention arm reported high overall satisfaction with the PDA tool: the mean overall satisfaction score was 4.2/5.0 (standard deviation 0.52). Responses for other questions are shown in Figure 2, showing high satisfaction with the content and format of the PDA. Based on open text responses, perceived strengths of the PDA included having a helpful summary of the evidence compiled by experts in a user-friendly format and gaining a greater understanding of the risks of non-medicated depression. Perceived limitations included the lack of data on longer term child outcomes, lack of data on the relative effects of different antidepressants, and conflicting information being given by the PDA and the clinician, or by different clinicians.

In terms of acceptability to clinicians, only six clinicians returned the questionnaires, so only qualitative findings are reported. Positive comments included a perceived benefit in terms of patient empowerment and knowledge; concerns included increased patient anxiety following PDA use; and suggestions for change included clinicians having access to the PDA.

### Clinical outcomes

Maternal clinical measures of decisional conflict, depressive symptoms, anxiety symptoms, and knowledge are summarised in Table 2 and Supplementary Figure 1(a–c). Decisional conflict decreased significantly between baseline and t1 in both the intervention and control arms. ANCOVAs of the potential effectiveness of the PDA showed that there was a trend in the desired direction in the DCS score in the PDA versus control arms (regression coefficient -3.5, 95% CI = -12.6 to 5.6). Similar findings were observed for depressive and anxiety symptoms at t2 (see Table 3). No harms were reported.

**Table 2.** Efficacy outcomes: summary scores at baseline and follow-up

		Mean (SD)	Difference in means (95% CI)
Measure (score range)	Study group	Baseline score^a^	t1 score^a^	t2 score^b^	Difference between t1 and baseline score	Difference between t2 and baseline score
							DCS (0–100)^c^	PDA	53.2 (11.5)	32.2 (16.4)	NA	-21.0 (-26.9 to -15.0)	NA
	No PDA	56.7 (16.6)	37.5 (17.0)	NA	-19.2 (-27.3 to -11.0)	NA
EPDS (0–30)	PDA	14.5 (7.1)	12.8 (6.6)	9.0 (4.8)	-1.7 (-4.0 to 0.61)	-4.9 (-7.9 to -1.9)
	No PDA	12.5 (6.2)	10.3 (5.9)	9.7 (5.5)	-2.2 (-4.9 to 0.47)	-2.7 (-5.2 to -0.27)
STAI-state (20–25, 27–80)	PDA	44.0 (15.2)	44.8 (15.5)	38.8 (13.7)	0.74 (-3.5 to 5.0)	-4.2 (-11.8 to 3.5)
	No PDA	41.8 (12.8)	38.1 (12.3)	42.2 (12.6)	-3.7 (-9.0 to 1.6)	0.35 (-5.6 to 6.3)
Knowledge questionnaire (0–16)	PDA	11.3 (2.2)	12.5 (1.7)	NA	1.2 (0.23 to 2.1)	NA
	No PDA	11.0 (1.5)	12.2 (1.9)	NA	1.1 (0.33 to 1.9)	NA

^a^ *N* for baseline and t1 (4-week follow-up) for all outcomes: PDA = 23, no PDA = 23. ^b^ *N* for t2 (long-term follow-up): for EPDS scores PDA = 21, no PDA = 23; for STAI scores PDA = 20, no PDA = 23.

CI = confidence intervals. DCS = decisional conflict scale. EPDS = Edinburgh postnatal depression scale. NA = not applicable. PDA = patient decision aid. STAI = state-trait anxiety inventory.

**Table 3.** Efficacy outcomes: analyses of covariance

		ANCOVA^a^ for t1 score	ANCOVA^a^ for t2 score
***n***	**Regression** **co-efficient**	**Partial eta squared** ^**b**^	***n***	**Regression** **co-efficient**	**Partial eta squared**
	**(** **95%** **CI** **)**	**(** **95%** **CI** **)** ^**c**^		**(** **95%** **CI** **)**	**(** **95%** **CI** **)** ^**b**^
DCS	PDA	23	-3.5 (-12.6 to 5.6)	0.014 (0.000 to 0.143)		—	
	No PDA	23					
EPDS	PDA	23	1.4 (-1.6 to 4.5)	0.020 (0.000 to 0.158)	21	-1.3 (-4.1 to 1.5)	0.021 (0.000 to 0.163)
	No PDA	23			23		
STAI (state)	PDA	23	5.1 (1.0 to 11.3)	0.062 (0.000 to 0.230)	20	-3.8 (-11.4 to 3.8)	0.025 (0.000 to 0.174)
	No PDA	23			23		
Knowledge questionnaire	PDA	23	0.13 (-0.86 to 1.1)	0.002 (0.000 to 0.090)		–	
	No PDA	23					

^a^ANCOVA model of effect of allocation, adjusted for baseline score of the relevant outcome measure. ^b^Standardised effect sizes were estimated using partial eta squared (by convention, values of 0.01, 0.09, and 0.25 indicate small, medium, and large effect sizes respectively). ^c^The lower limit of the confidence interval was estimated as 0.0 for all analyses.

ANCOVA = analysis of covariance. CI = confidence intervals. DCS = decisional conflict scale. EPDS = Edinburgh postnatal depression scale. PDA = patient decision aid. STAI = state-trait anxiety inventory.

## Discussion

### Summary

This pilot RCT of a PDA for antidepressant use in pregnancy showed that the trial protocol was feasible, with high follow-up rates. Recruitment strategies and sources had to be expanded beyond local primary care and maternity and psychiatric services, to include other maternity services and national online self-referral, to meet recruitment targets. There was high overall patient satisfaction, with the vast majority of women agreeing that the PDA was useful and that they would recommend it to others. By design, this pilot RCT was not powered to detect differences in maternal clinical outcomes, but findings from this pilot are consistent with the intervention having a small effect size on decisional conflict at 4 weeks post-randomisation and a small effect size on depressive and anxiety symptoms at longer-term follow-up in the desired direction.

### Strengths and limitations

Strengths of this pilot RCT include the identification of feasible methods of recruitment (once recruitment strategies were expanded) and high follow-up rates. There were high rates of participant acceptance of study method and satisfaction with the PDA. A further strength was successful collaboration with decision aid experts in Canada, where there is a strong clinical tradition of developing quality-assured decision aids (following the International Patient Decision Aids Standard guidelines)^13^ and routine clinical use of PDAs.

Women were recruited via maternity services in several regions and via national direct advertising to patients, so pilot recruitment methods for these settings are likely to be generalisable to the UK setting. However, recruitment from primary care and psychiatric settings was limited to a local area, with low recruitment rates via these routes. Prior to a definitive trial, it would be useful to clarify referral barriers within the latter settings. In this pilot, there was limited success in obtaining feedback from clinicians regarding the acceptability of the intervention, receiving only six completed surveys, and it is therefore not possible to draw any conclusions about clinicians’ attitudes towards the PDA. More generally, the PDA was targeted at patients, with online access given to patients but not clinicians. In the UK setting, it may be important to make the PDA available to both clinicians and patients, with a focus on primary care settings, since antidepressants are largely prescribed and managed in this setting. The decision aid could be used as a shared tool for use in consultation instead of, or as well as, women having access to this at home. This would encourage shared decision-making, reduce the likelihood of women receiving conflicting information from the decision aid and their healthcare professional, and ensure that clinicians also have access to the latest evidence base. The potential importance of this is underlined by feedback from some participants that they received conflicting information from their clinician and the PDA.

A qualitative study exploring decision-making about antidepressant use in pregnancy was conducted in Canada and fed into the development of the decision aid.^8^ However, qualitative work in the UK setting would be valuable to explore how decision-making occurs in practice and how a decision aid could provide support in this area. This work should include interviews with women making this decision, partners and/or family members, and healthcare professionals (including GPs, midwives, and psychiatrists). Findings could be used to ensure that the PDA is optimised for use in the UK. Developments to the PDA could be codesigned with experts by experience and professional stakeholders, which could improve uptake by clinicians and ensure that the PDA addresses women’s needs. The current study did not systematically collect information on how women used information from the PDA in their consultations with healthcare professionals, and whether they shared the PDA or information from it with significant others, including their partner. Future evaluations should collect this data to improve knowledge of how PDAs are used in practice.

### Comparison with existing literature

To the authors’ knowledge, there are no published trials on decision aids for psychotropic use in pregnancy. A recent Cochrane Review that evaluated 105 RCTs of decision aids across different treatment and screening decisions found moderate to high quality evidence that decision aids increased people’s knowledge about treatment options, helped them to have more accurate expectations of benefits and harms of treatment, and helped them to participate more in the decision-making process.^23^ A systematic review of decision aids specifically for pregnant women found evidence from 10 trials that decision aids increased knowledge and decreased decisional conflict and anxiety.^24^ In the UK, NICE recommends the use of high-quality decision aids to help patients make preference-sensitive medications decisions, but decision aids are not routinely used in clinical practice.^25^ This is partly because of the limited availability of evaluated decision aids and research into barriers for their use, so this pilot trial and future related full-scale trials would address an important gap.

### Implications for research and practice

Findings from this pilot suggest that it would be feasible to recruit women into a definitive trial for this PDA using the study protocol, and that the PDA has high acceptability for study participants. The findings show that decisional conflict decreases significantly over time regardless of intervention, but that anxiety levels were high at baseline and remained high at follow-up. Although decisional conflict is often used as a primary outcome for decision aids, in this patient population anxiety and mood symptoms may be equally important and may be more valuable outcome measures in a definitive trial, including longer-term symptom follow-up in late pregnancy and postnatally. Additionally, this pilot only included women with moderate-to-high levels of decisional conflict at baseline. If an alternative primary outcome is used, future studies should consider including women regardless of baseline decisional conflict level, as all women need access to evidence-based information to enable them to make fully-informed decisions. A key inclusion criterion for this pilot was recommendation of antidepressants for treatment of depression during pregnancy, but in this study sample current anxiety rates were higher than current depression rates, and the two are often comorbid. In a definitive trial, it may be useful to expand recruitment to women with anxiety or depressive disorders, although the PDA would need to be developed to include information about anxiety disorders during pregnancy, and the potential risks and benefits of antidepressants as a treatment for anxiety.

The reported conflict between information provided by the PDA and some clinicians, and the limited ability to recruit through primary care and to obtain clinician feedback, suggests the need for greater involvement of clinicians, particularly GPs, in the design and delivery of the RCT, as described above. In order to ensure that the PDA remains up to date with the evidence base, and that expert input into this is adequately costed, it would be important to assess cost-effectiveness in a future definitive trial.

This UK-based pilot RCT was an adaptation of a pilot RCT in Canada, where this PDA was originally developed. The ability to successfully adapt and pilot this PDA across international borders points to the potential for further international collaboration using online interventions. It may be necessary, however, to further adapt the way the PDA is used in practice within the context of routine NHS care.

## Trial registration number

The trial protocol was registered at clinicaltrials.gov (reference number: #NCT02492009)
